# Survey on current clinical practice in geriatric oncology: the individual experience in five European Cancer Centers

**DOI:** 10.1007/s41999-024-01041-7

**Published:** 2024-10-23

**Authors:** M. Javier-González, R. Boulahssass, L. Dal Lago, N. M. González-Senac, S. Nair, M. Vetter

**Affiliations:** 1 Department of Oncology and Hematology, Kantonsspital Baselland Liestal, Liestal, Switzerland; 2 Geriatric Coordination Unit for Geriatric Oncology (UCOG), Centre Hospitalier Universitaire de Nice, University of Côte d’Azur, FHU OncoAge, Nice, France; 3 Comprehensive Cancer Center, Delta Hospital, Chirec Hospital Group, Brussels, Belgium; 4 Geriatrics Department, Biopathology of Aging Group, Hospital General Universitario Gregorio Marañón, Health Research Institute Gregorio Marañón (IiSGM), Madrid, Spain; 5 Department of Elderly Medicine, Leeds Teaching Hospital Trust, Leeds, UK

**Keywords:** Geriatric oncology, Interdisciplinary teams, Models of care, Interprofessional collaboration

## Abstract

**Purpose:**

To gather information from clinicians on how geriatric oncology models of care have emerged in different European countries and describe current practice in this clinical area.

**Methods:**

A semi-structured online interview was performed, exploring aspects related to implementation, perceived quality of care, and professional satisfaction.

**Results:**

The centers participating in this interview showed significant differences in terms of resource allocation, team members, components of the comprehensive geriatric assessment (CGA), and CGA-driven interventions. High levels of professional satisfaction were expressed by all participants. This was deemed a consequence of a perception of increased quality in the provision of care and enhanced educational and academic opportunities.

**Conclusion:**

Interdisciplinary models of care in geriatric oncology, regardless of implementation details, seem to provide grounds for increased professional satisfaction and perception of better provision of quality of care. These characteristics could support promoting and further developing similar collaborations on a wider scale.

**Supplementary Information:**

The online version contains supplementary material available at 10.1007/s41999-024-01041-7.

## Key summary points

**Aim:**

To gather information from clinicians on how geriatric oncology models of care have emerged in different European countries and describe current practice in this clinical area.

**Findings:**

Interdisciplinary models of care in geriatric oncology are perceived to present benefits in terms of quality of care and increase professional satisfaction among team members.

**Message:**

Supporting models that promote Person-Centered Care and professional satisfaction could represent an opportunity to retain healthcare workforce.

**Supplementary Information:**

The online version contains supplementary material available at 10.1007/s41999-024-01041-7.

## Introduction

Since the early 1980s, specialists in oncology have recognized a growing incidence of cancer among older adults, as well as particular characteristics of this population, including frailty, that increase the challenge of selecting therapeutic approaches. The term geriatric oncology appeared in the mid-1980s and education and research in this field have been endorsed by the American Society of Clinical Oncology (ASCO) since 1988 [1].

Thereafter, efforts have been made to augment the inclusion of older adults in oncological clinical trials, thus improving evidence to support certain therapies. Nevertheless, there is still an underrepresentation of older adults in cancer trials with a participation of 10–25% of people 70 years and older, these participants typically have fewer functional impairments or comorbid conditions than the average older patient in clinical practice [2].

International societies are being encouraged to recommend geriatric assessment (GA) for all older adults with cancer [3, 4], and there seems to be a commitment to integrate geriatric evaluation into oncological decision-making and treatment guidelines, as well as increasing their participation in research.

Simultaneously, inter-professional collaborations in geriatric oncology have grown, driving the implementation of services where interdisciplinary teams make joint treatment decisions for older adults with cancer [5]. Still, centers offering geriatric oncology are frequently restricted to tertiary academic hospitals resulting in large inequalities in the availability of clinical, educational, and research initiatives across different regions [6].

Many different models in geriatric oncology are implemented across numerous centers [5, 7]. These models differ in many aspects including—but not limited to—specialties of the team members, the setting in which they take place, frequency of assessments and interactions, tools used as part of the comprehensive geriatric assessment (CGA), and the interventions resulting from it. Agreement has been reached regarding the GA domains to evaluate [8]; however, a single optimal tool to perform this assessment has not been defined so far [3].

To explore different models of care in geriatric oncology in various European countries, we designed and conducted a written interview with colleagues active in this field, whose main professional activity takes place in tertiary centers in five different European countries that provide geriatric oncology care. The main objective was to gather information on how these models of care have emerged and to describe current practice.

## Methods

A semi-structured written interview with open-ended questions was designed to explore qualitative aspects of the implementation of geriatric oncology services in different European centers.

The interview comprised identifying questions including clinical specialty, workplace, and role within their respective teams. Subsequent questions had the objective of gathering information on resources, patient selection for inclusion in the interdisciplinary model of care, assessments and tools included in their CGA, as well as the type of interventions performed. Information regarding the style and means of communication within teams, and finally, questions regarding team members’ and patients’ satisfaction were requested. The interview questions are shown in online Appendix A.

Due to the exploratory nature of the initiative, a limited number of potential participants was identified to facilitate data presentation. Potential participants were selected by a search of the list of national representatives of the International Society of Geriatric Oncology (SIOG) and the Young SIOG Interest Group. Initially, six potential participants from six different European countries were selected and approached via email to request written or verbal agreement to participate in the interview. A positive answer was received from five of the six candidates.

Subsequently, the written interview was conducted online using Google Forms. A reminder was sent after 4 weeks. After 6 weeks, the interview was closed. The definitive answers were collected and summarized by topic in a descriptive manner. The following steps included a review of the written summary from each participant to corroborate the accuracy of the information and receive approval for submission.

## Results

### Experiences by country

A summary of responses according to topic addressed is presented in Table 1.

**Table 1:** Summary of answers

	Belgium	France	Spain	Switzerland	United Kingdom
Initiative	Oncologists	Oncologists	Surgeons/geriatricians	Oncologists	Surgeons/geriatricians
Age of screening	70 yo	70 yo	74 yo	70 yo	75 yo
Selection tool	G8	G8 Clinical decision	Age	G8 Clinical decision	Age CFS Clinical decision
Team members	Geriatrician Oncologist Oncology nurse Occupational therapist Radio-oncologist	Geriatrician Oncologist Onco-geriatric nurse Psychologist Social worker Secretary	Geriatrician General surgeon Geriatric nurse Surgical nurse Physical exercise expert	Geriatrician Oncologist Onco-geriatric nurse	Geriatrician Onco-geriatric nurse Advanced clinical practitioner
Geriatric specialist participation tumor board	No	Yes	No	Yes	No
Frailty assessment	Edmonton scale	Balducci criteria	Fried’s criteria, CFS, G8, SPPB	G8	CFS
CGA—domains assessed	Cognition Frailty ADLS	Comorbidities Cognition Frailty ADLS Nutrition Depression Anxiety Gait Sensorial function QoL Survival scores	Comorbidities Cognition Frailty ADLS Nutrition Depression Anxiety Physical measures Medication Social status QoL	Comorbidities Cognition ADLS Nutrition Depression	Cognition Frailty ADLS Delirium Mental capacity
Interventions	Medication review Pre-habilitation Outpatient clinics Follow-up visits	Medication review Treatment modification Nutritional advice Physiotherapy Management of comorbidity	Medication review Nutritional advice Physiotherapy Cognitive therapy Coordination of care Management of comorbidity	Medication review Blood pressure control Nutritional advice Falls prevention	Medication review Coordination of care Management of comorbidity Suitability for tests/treatment Escalation plans
Professional satisfaction	+	+	+	+	+

*ADLS* Activities of Daily Living, *CFS* Clinical Frailty Scale, *CGA* Comprehensive Geriatric Assessment, *QoL* Quality of Life, *SPPB* Short Physical Performance Battery, yo years old

#### Belgium

Participant: oncologist, team leader.

##### Origin

The idea of a geriatric oncology collaboration originated with a project proposal in a private general hospital from a medical oncologist to perform a pilot phase of Geriatric Oncology care. The medical oncologist had extensive experience from an academic hospital in the same domain.

##### Resources

The resources to perform clinical activities were provided by the institution with the support of the Geriatrics and Oncology departments.

A geriatrician, two oncology nurses, an occupational therapist, a radiation oncologist, and a gastrointestinal and breast cancer medical oncologist comprise the team, as part of their clinical activities within the center.

An interdisciplinary meeting takes place every 2 weeks to discuss cases with a geriatric assessment (GA).

##### Patient selection

Patients 70 years of age and older are identified either at initial cancer diagnosis, disease progression, or recurrence, and then referred to the geriatric oncology team from the outpatient clinic. A Geriatric-8 (G8) is performed and patients with a score of ≤ 14 will be classified as frail and become candidates to be included for a complete CGA before initiating treatment.

##### Comprehensive geriatric assessment

The GA is performed by an oncology nurse or an occupational therapist, and systematically documented in the electronic health records (eHR) of each patient. Functional and cognitive parameters, as well as frailty, are evaluated. The Edmonton Scale will be used for frailty assessment.

##### GA-driven interventions

After this, a specialist geriatrician proposes guided interventions to address identified deficits. Interventions include medication review, visits to the geriatric outpatient clinic, pre-habilitation, and follow-up visits.

##### Interprofessional communication

Interventions are delivered in the form of suggestions rather than an active approach, despite this, there is the perception within team members that this collaborative model drives modifications in the therapeutic approach in the older person with cancer.

##### Patient- and team satisfaction

The participant in this interview perceives a clear increase in the team- and patients’ satisfaction since the introduction of this collaborative model, though this has not yet been formally evaluated. The participant “could not imagine going back” to her previous working model and estimates that her team members would give a similar answer if asked.

At present, classical medical outcomes, patient-related outcome measures (PROMs), patient satisfaction, and health-related quality of life (HR-QoL) are not yet systematically recorded, but projects in this regard are underway.

#### France

Participant: geriatrician, team leader.

##### Origin

The interest in starting a geriatric oncology collaboration originated within the oncology team of this university hospital in the year 2000.

##### Resources

From the beginning, both geriatrics and oncology departments have shared the costs of implementation, as well as the provision of staffing and environmental resources for the geriatric oncology team.

Since its start, the geriatric oncology collaboration in this hospital has explored different models such as outpatient specialized clinics, sharing committees, and research. Nowadays, the service is developing a digital patient platform with the participation of specialized nurses.

The geriatric oncology team in this center comprises two geriatricians, an oncologist, specialized nurses, a psychologist, a social worker, and a secretary.

##### Patient selection

Referrals for a geriatric oncology assessment originate from outpatient oncological/surgical consultation, after identification in one of the weekly tumor boards of this center, or from patients included in specific research projects.

Older patients with a cancer diagnosis are identified for eligibility using a G8 questionnaire. However, some patients are referred for an assessment regardless of the results of the G8 if the treating oncologist, surgeon, or radiotherapist considers them candidates.

##### Comprehensive geriatric assessment

The CGA in this center includes assessment of comorbidity, activities of daily living (ADLs), cognition, gait, and sensorial function, nutrition, anxiety and depression, and QoL, as well as survival scores. Frailty is evaluated using the Balducci Frailty Criteria [9]. The assessments are standardized and used for clinical and research purposes.

Doctors and nurses in this team share the responsibility of performing a CGA, as well as documenting its results. The results are reported numerically and sent to the referring physician right after their performance in the outpatient clinic.

##### GA-driven interventions

The interventions derived from this CGA include 12 different domains, among which the most frequent are nutritional advice, physiotherapy interventions, management of comorbidities, and modification of treatment and medication [10].

The geriatric oncology team actively facilitates these interventions by direct modification of medication, direct referrals to physiotherapists, or nutritional advice. This implies sharing responsibility for the patient among all team members.

Since its conception, the service has actively participated in clinical research for geriatric oncology for which they systematically record and analyze their performance, including the Provence-Alpes-Cote d’Azur (PACA) Cohort. The PACA cohort is a database created in 2011 to upgrade care, research, and teaching in geriatric oncology in the Southeast of France and has included more than 7000 patients. Interventions derived from the CGA in this center have demonstrated driving changes in the therapeutic approach in 22% of the patients, as published in their article “The desire to better understand older adults with solid tumors to improve management” [10].

##### Interprofessional communication

Communication within the team takes place through different means, ranging from e-mail to verbal communication and personal meetings.

In this model of care, there is an active participation of a geriatrician on the hospital’s tumor boards. The geriatricians directly express their criteria and recommendations making them active participants in oncological therapeutic decision-making.

##### Patient- and team satisfaction

Since working in this model, the team members perceive an improved communication and flow of information. This has an impact on the morale of the team, who feel they are helping patients and are very proud of their work. The participant also perceives an increase in her levels of job satisfaction, through reduced stress, the impression of delivering higher quality of care, and being more helpful to patients. She believes other team members would have a similar answer to this question. Although not measured yet, the participant considers that patients would also perceive improvements.

With the utilization of the digital platform, the team foresees including measures of QoL, patient satisfaction as well as PROMs in the near future.

#### Spain

*Participant*: geriatrician.

##### Origin

Around 2020, the geriatricians in this university hospital had the idea of developing a project with the general surgeons to improve the provision of care among older adults with a surgical indication for colorectal cancer. The initial idea was to provide peri-operative care including pre-habilitation, as well as follow-up after surgery and long-term follow-up during systemic treatment.

##### Resources

The geriatric service could appoint a consultant geriatrician and a nurse practitioner for the implementation of the project. Funding from a randomized control trial (RCT) in geriatric oncology has provided the possibility of including a physical exercise expert in the team.

At present, general surgeons, a surgical nurse practitioner, a geriatric nurse practitioner, two consultant geriatricians, and a physical exercise expert compose the geriatric oncology team in this center. There is occasional participation of a psychologist.

##### Patient selection

Referrals originate from the general surgery outpatient clinic after an indication for surgery has been established.

Patients 75 years of age and older diagnosed with a colorectal neoplasm and with a surgical indication are candidates for a complete CGA, carried out by the geriatrics team in the context of a geriatric oncology outpatient clinic.

##### Comprehensive geriatric assessment

The CGA includes functional and physical measures, measures of comorbidity, medications, mental status including cognition, depression and anxiety, frailty, nutrition, social status, and QoL. Frailty is assessed with Fried’s criteria, the Clinical Frailty scale (CFS), the G8 questionnaire, and the Short Physical Performance Battery (SPPB).

##### GA-drive interventions

The interventions resulting from this CGA include management of comorbidities, medication review and adjustments, coordination of care, and nutritional, physical, and cognitive intervention, among others.

There is the perception that geriatric interventions drive changes in the therapeutic approach to patients diagnosed with colorectal cancer who are surgical candidates. These modifications are currently being systematically recorded and analyzed, along with medical outcome measures, patient satisfaction, and QoL.

##### Interprofessional communication

The team members use direct verbal communication as the main means of communication, as well as documentation in the medical notes. All domains are systematically documented in the patient’s records and a structured data collection form for research purposes by the geriatric nurse practitioner or the consultant geriatrician.

At present, there is no participation either from the geriatrician or the geriatric nurse in the tumor board.

##### Patient- and team satisfaction

There are clear perceived benefits of this collaboration among team members. The participant has experienced positive changes in job satisfaction, and continuing education since working in this interdisciplinary model. The same experience has been expressed by the rest of his team members in a recent joint meeting.

#### Switzerland

*Participant*: oncologist, project- and department lead.

##### Origin

After being appointed as clinical lead of Hematology and Oncology in his center, the participant had the initiative to start a geriatric oncology collaboration. In this teaching hospital, more than 700 patients are diagnosed with a new cancer each year, of which around 70% are 65 years of age or older. This project is still in the first phases.

##### Resources

To date, this collaboration has been granted a 10% full-time equivalent (FTE) for a geriatric oncology nurse. However, the estimated needs are much higher.

The geriatric oncology team includes a specialist in oncology, a specialist in geriatrics, and a geriatric oncology nurse. In the future, they expect to involve other healthcare professionals including—but not limited to—radio-oncologists, clinical pharmacists, palliative care specialists, psychologists, spiritual care, physiotherapists, and dieticians.

##### Patient selection

Patients 70 years and older diagnosed with a new cancer are screened using a G8 test for eligibility for a complete CGA. Those scoring ≤ 14 points are identified as frail and discussed in an interdisciplinary geriatric oncology board. In this discussion, the opinion of the patient’s primary care physician is also explored.

The possible outcomes are either referral to the geriatric oncology outpatient clinic for a complete CGA, treatment optimization in the oncology clinic—including chemotherapeutic dose reduction, therapeutic interventions from physiotherapists, psychologists, and dieticians, or Best Supportive Care (BSC).

##### Comprehensive geriatric assessment

The CGA is expected to be based on the latest ASCO/SIOG advice [3] and adaptations will be done according to the locally used assessment tools. Functionality is assessed using the functional independence measure (FIM), comorbidities using the Charlson comorbidity index (CCI) or the cumulative illness rating scale-geriatric (CIRS-G), cognition using the mini-mental status examination (MMS), depression using the ultra-short form of the geriatric depression scale (GDS), and nutrition using a nutritional risk score (NRS). Other assessments of performance such as Time-Up and Go, and Tinetti are still under review.

Given the current resource availability, an appointed geriatric oncology nurse and a junior doctor on their geriatric rotation are responsible for performing and documenting the results of the assessments in the eHR and in an electronic database for research purposes.

##### GA-driven interventions

The expected interventions from the geriatric oncology team include medication review, blood pressure control, nutritional optimization, and interventions to prevent falls.

The geriatrician will actively modify medications and prescribe physiotherapy and physical activity. These interventions are still to reach internal agreement among team members.

There is the perception, that once in place, the geriatric oncology interventions will impact the therapeutic approach. A systematic analysis of this impact is going to be performed with the aid of the research database.

There are numerous expected benefits from this collaboration ranging from an improvement in the quality of care for the older patient with cancer, avoiding under- and overtreatment through the use of international recommendations in geriatric oncology, and enhancement of research and educational activity in this field in the region. The team also expects better communication among the specialists involved.

##### Interprofessional communication

After the geriatric oncology outpatient clinic, the referring physician is going to receive a letter from the team with the results of the assessments and proposed interventions.

##### Patient- and team satisfaction

Since the project is in its first steps, there has not been any perceived changes in terms of job satisfaction among team members. The team is, however, very optimistic about this interdisciplinary collaboration.

#### United Kingdom

*Participant*: geriatrician.

##### Origin

The idea of a geriatric oncology collaboration originated from a joint interest of the surgical-oncology and geriatric teams of this large university hospital. Forty percent of cancer patients in the area present with some degree of frailty, in view of this, both teams considered there was a clear indication for the participation of a geriatrician in the optimization of peri-operative management of people older than 75 years of age.

##### Resources

From its beginning, the service was provided with limited economic resources, funding constraints being the main challenge to further development of this collaboration. At present, efforts are being made to train allied health staff to deliver the service and ensure cost-effectiveness.

Currently, the geriatric oncology team in this center comprises a specialist nurse, an advanced clinical practitioner, and a consultant geriatrician, who provide support to the outpatient clinic for gastrointestinal tumors.

##### Patient selection

Referrals originate from primary care through the 2-week cancer pathway. The 2-week cancer pathway is a scheme within the National Health Services (NHS) that ensures that patients presenting with symptoms suggesting cancer are seen in specialized clinics within two weeks for further clarification [11].

The growing volume of referrals from primary care poses the issue of determining the adequate service to see these patients (i.e., urgent referrals vs fast-track cancer clinics), as well as ensuring continuity of care to provide a CGA and shared decision-making. There is a perspective to enhance this by including patients awaiting and undergoing medical or surgical treatment.

##### Comprehensive geriatric assessment

The CGA in this center includes an evaluation of physical function, as well as a CFS to determine the presence of Frailty, a 4AT Test to detect Delirium, a Montreal cognitive assessment (MoCA) when cognitive impairment is suspected, and capacity assessments if considered appropriate.

Patients with a CFS of ≥ 6 defined as being moderately frail are eligible for this service.

##### GA-driven interventions

After the initial CGA, interventions include optimization of medical conditions, medication review, and discussions regarding escalations of care, including resuscitation where required. The team also makes decisions regarding the suitability of the patient for invasive tests and/or treatment. This is actively communicated to the patients, next-of-kin, and other specialists.

Since its beginning, the geriatric oncology team has significantly transformed the practice in the outpatient clinic for patients with suspected gastrointestinal cancer. Treatment modalities are discussed with the patient and relatives, including conservative and palliative approaches. Variables such as QoL and “what matters most” to the patient, as well as their prognosis adjusted by frailty status, are evaluated and discussed with the patient.

The team ensures that patients identified as suitable after a CGA undergo appropriate testing and treatment regardless of age. On the other hand, the team informs patients considered unsuitable for active treatment about other therapeutic possibilities and supports decisions beyond classic active oncological treatment.

This team systematically records and analyses the outcomes of their patients through follow-up. This facilitates regular audits. The following outcome measures are used: 1-year mortality, patient satisfaction, costs of treatment, and recurrence within 12 months.

In their experience, a high number of patients die within 18 months due to chronic medical conditions different than cancer. In their opinion, this fact supports the existence of a geriatric oncology team.

##### Interprofessional communication

All team members are responsible for recording CGA variables on the eHR. The geriatric oncology team communicates the results of the assessments from the CGA, as well as their clinical criteria and advice through clinic letters to the patient and primary care.

At present, there is no participation of the geriatric oncology team in tumor boards, however, this is currently been discussed.

##### Patient- and team satisfaction

Many benefits from the collaborative model of care are perceived. They see an improvement in communication and shared decision-making, which has a positive impact on inter-professional relationships. Another benefit is an increase in clinical and scientific collaborations.

As a geriatrician, the participant recognizes advantages in terms of continuing medical education.

In addition, the team is involved in education and support to junior doctors, advanced practitioners, registrars, primary care physicians, and other specialists.

From an administration perspective, the team has been able to demonstrate cost-effectiveness by reducing expenditures in comparison to a standard surgical outpatient clinic.

There are, however, significant economic constraints, that prevent the expansion of the service. The team is working on engaging the hospital executive board through organized sessions to obtain economic and staffing resources.

## Discussion

With this interview, we intended to explore and describe how geriatric oncology collaborations have emerged and function in a limited number of European countries. Among participating centers, oncology, general surgery, and geriatrics professionals have designed initiatives to implement geriatric oncology collaborations that differ in aspects ranging from the appointment of resources, healthcare professionals involved, components of the CGA and tools used, and means of communication and intervention.

Lack of economic support from healthcare systems was perceived to be the main constraint to further developing these collaborations by our participants. In a systematic realist review, McKenzie et al. enumerate several barriers to implementing GA into oncological practice, among them, limited resources, absence of funding, limited workload capacity, and uncertain practicalities [12], factors similar to those expressed by the participants in this interview. At the same time, organizational factors and the engagement of multiple stakeholders, including healthcare professionals and policymakers, play a determinant role in the implementation of GA in everyday practice [13]. ASCO’s latest 5-year strategic plan for geriatric oncology proposes a pragmatic integration of GA into everyday practice [14], designed around the numerous challenges mentioned above. The experiences reported by the participating centers seem to display this pragmatism and resilience.

Various assessment tools are used to perform the CGA among our participants, which may be explained by the lack of consensus regarding the optimal tools to include in a CGA in oncology. This variability could potentially have an impact on research initiatives and limit comparisons in the scientific field. Nevertheless, frailty, functional status, and cognition are being assessed by all participants. A Consensus of Geriatric Oncology Experts proposes the inclusion of measures of physical function and performance, comorbidity and polypharmacy, cognition, nutrition, psychological status, and social support [8]. All these aspects are evaluated as part of the practical geriatric assessment (PGA) [3, 15], recommended by the ASCO oncology guidelines. This tool could present another opportunity to standardize initial assessments, facilitate communication and interpretation of results, as well as tackle barriers such as time and resource constraints.

Overall, the main novelty introduced by this interview is the focus on the perceived quality of care and physicians’ satisfaction. Participants in this interview perceive an improvement in the provision of care enabled by an interdisciplinary model. In addition, they express personal and professional satisfaction, which may be a consequence of enhanced educational and academic opportunities, professional interaction among people with a common interest, better communication, and possibly a feeling of shared responsibility and success.

Physicians’ well-being is often a neglected subject that cannot be ignored given foreseeable shortages of healthcare workers in the next years [16]. One of the key findings of a previous study endorsed by the American Medical Association (AMA) is that the perception of providing high-quality care, as well as fostering collegiality, increases satisfaction [17]. In addition, providing care by an interdisciplinary team seems to increase the effectiveness, affect work efficiency, and improve professional relations of medical staff [18]. Emphasizing models of care that provide its members with opportunities for professional development as well as open interaction and feeling of recognition could represent an incentive to increase collaborative work and support the development and propagation of interdisciplinary models of care.

We acknowledge the limited number of respondents in this interview, as well as the possible introduction of a selection bias. However, we hypothesize that similar answers could be received if a survey on a larger scale was to be performed. This interview could open opportunities for the development of similar initiatives. We believe that an international structured survey on a global scale is warranted to further explore physicians’ and patients’ perceptions and satisfaction, as well as health-economic variables pertaining to models of care in geriatric oncology. Such studies could further demonstrate the benefits of collaborative approaches in geriatric oncology and support the expansion of these models.

The geriatric oncology services participating in this interview have been independently created without specific implementation guidelines, and therefore, vary significantly. However, they share fundamental similarities such as their vision of improving the quality of care for the older person with cancer and creating a favorable professional environment for their participating agents.

## Supplementary Information

Below is the link to the electronic supplementary material.

Supplementary file1 (DOCX 15 KB)

## Data Availability

The data supporting this study’s findings are available from the corresponding author upon reasonable request.
