# Supplementary material for: Survey on current clinical practice in geriatric oncology: the individual experience in five European Cancer Centers
Source: Eur Geriatr Med. 2024 Oct 23;16(1):125–33. doi: 10.1007/s41999-024-01041-7 (PMC11850469; doi:10.1007/s41999-024-01041-7)
Supplement: Supplementary file 1 — Supplementary file1 (DOCX 15 KB) [file 41999_2024_1041_MOESM1_ESM.docx]

| Appendix A: interview questions | |
| --- | --- |
| Topic | Questions |
| Origin of the geriatric oncology collaboration | How did the interest in starting an onco-geriatric collaboration originate in your work center? |
|  | Who came up with the initiative? (specialists, other members of the Multidisciplinary Team, administrators) |
| Resources | With which initial resources was this collaboration provided? Please, specify the type of resources (staffing, environmental, economic) |
|  | How have these resources developed up to this point in time? Are there any further foreseen developments in the plan? If yes, could you specify? |
|  | Which members compose your onco-geriatric team? Please include therapists, psycho-oncologists, volunteers, and all that apply in your description. |
|  | Do you have a dedicated area in the hospital for your onco-geriatric collaboration? If not, where do the assessment and professional exchange take place? |
| Patient selection | Which criteria do you use to determine whether a patient is going to be seen by your transdisciplinary team? |
|  | What type of patient do you include in this collaboration? (patients presented in tumour-board, patients seen in outpatient clinics, patients from emergency departments, others) |
| Comprehensive Geriatric Assessment | Which assessments are included in your Comprehensive Geriatric Assessment? |
|  | Is there structured/systematic documentation of frailty status, measures of function, and/or measures of cognition among your assessments? |
|  | If frailty is used: which tool/s or frailty criteria? |
|  | Which member/s of the team is/are in charge of performing and documenting these assessments? |
|  | How are the results documented? |
| Interprofessional communication | How do the members of the team communicate with each other? (Meetings, direct verbal communication, direct written communication through emails, discharge letters, and others) |
|  | Do you have the participation of a geriatrician or a geriatric nurse in your tumor-board? |
|  | To which level does the geriatrician in your team participate in onco-geriatric decision-making? (direct opinion or indirectly through results of assessments) |
| Geriatric interventions | What type of interventions are performed by the geriatrician in your onco-geriatric team (medication review, communication and coordination of care, other)? Please specify. |
|  | What method of intervention is used by the geriatrician in your onco-geriatric team? (active, or passive through suggestions, others). |
|  | What is the perceived impact of this participation in oncological decision-making? Do these interventions drive changes or modifications in the therapeutic approach? |
|  | Does your team systematically record and analyze this impact or modifications in the therapeutic approach? |
|  | Is there a systematic measurement of outcomes (including medical outcomes but Patient Reported Measures, patient satisfaction and perceived Quality of Life)? |
| Patient and team satisfaction | Are there any perceived benefits of this collaboration among team members? |
|  | Are there any perceived benefits of this collaboration among patients? |
|  | Have you experienced any changes in terms of your own job satisfaction, continuing education, and levels of stress since working in such a model? |
|  | What do you think other members of the team would answer to this question? |
